# O.1.3-6 Using a socio-ecological approach to explore healthy lifestyle in elite sport: perspectives of athletes, coaches and managers

**DOI:** 10.1093/eurpub/ckad133.103

**Published:** 2023-09-11

**Authors:** Fabienne d'Arripe-Longueville, Aurélia Chrétien, Kevin Haffner, Meggy Hayotte, Marjorie Bernier, Anne Vuillemin

**Affiliations:** Université Côte d'Azur, LAMHESS, France; Université Côte d'Azur, LAMHESS, France; Université Bretagne Occidentale, CREAD, France; longuevi@univ-cotedazur.fr; Université Côte d'Azur, LAMHESS, France; Université Bretagne Occidentale, CREAD, France; longuevi@univ-cotedazur.fr; Université Côte d'Azur, LAMHESS, France

## Abstract

**Purpose:**

Elite athletes are continually subjected to a range of constraints specific to high performance, and these can have a negative impact on their health. Although many studies have explored the individual factors related to risky behaviors and disorders in elite sport contexts, and have provided prevention programs, few have focused on health promotion. Consequently, the interpersonal, institutional, and policy factors of the health-related behaviors of elite athletes are still poorly explored. Based on the socioecological model of health, this study aimed to identify the factors involved in the health-related lifestyle of elite athletes.

**Methods:**

Semi-structured interviews were conducted with 45 participants: athletes (N = 32; Mage=20.1, SD = 4.4), their coaches (N = 6; Mage=45.3, SD = 6.4) and managers of elite sport centers (N = 7; Mage=52.0, SD = 7.0). Interviews were audio recorded and transcribed verbatim. Both deductive and inductive thematic analyses were performed. This study was approved by the local Ethic Committee of the University.

**Results:**

Our results highlight the different levels of factors related to the health-related lifestyle of elite athletes, using the socioecological model, as perceived by athletes themselves, coaches, and managers. Regarding intrapersonal factors, resilience qualities and health literacy appeared as key factors to influencing the health-related lifestyle of elite athletes. At the interpersonal level, parents and coaches were considered as the main sources of educational support. Regarding institutional factors, the health policies of the elite sport centers were key factors in supporting the athletes’ healthy lifestyle. Similarities and specificities of the views of the different actors have been identified.

**Conclusion:**

These results show the interest of the application of the socioecological model in the elite sport domain. They encourage the development of health-promotion programs at different levels of intervention of the socioecological model, beyond traditional health prevention programs. In addition, this study suggests to consider the different perspectives of each actor to co-construct health promotion programs.

